# Urine hepcidin, netrin-1, neutrophil gelatinase-associated lipocalin and C-C motif chemokine ligand 2 levels in multicystic dysplastic kidney

**DOI:** 10.1590/2175-8239-JBN-2019-0022

**Published:** 2020-08-19

**Authors:** Nuran Cetin, Zeynep Kusku Kiraz, Nadide Melike Sav

**Affiliations:** 1 Eskisehir Osmangazi University, Faculty of Medicine, Department of Pediatric Nephrology, Turkey.; 2 Eskisehir Osmangazi University, Faculty of Medicine, Department of Biochemistry, Turkey.

**Keywords:** Hepcidins, Netrin-1, Multicystic Dysplastic Kidney, Child, Hepcidinas, Netrina-1, Rim Displásico Multicístico, Criança

## Abstract

**Introduction:**

Glomerular hyperfiltration may lead to proteinuria and chronic kidney disease in unilateral multicystic dysplastic kidney (MCDK). We aimed to investigate the urine neutrophil-gelatinase-associated lipocalin (NGAL), netrin-1, hepcidin, and C-C motif chemokine ligand-2 (MCP-1/CCL-2) levels in patients with MCDK.

**Methods:**

Thirty-two patients and 25 controls were included. The urine hepcidin, netrin-1, NGAL, and MCP-1/CCL-2 levels were determined by ELISA.

**Results:**

The patients had higher serum creatinine (Cr) levels, urine albumin, and netrin-1/ Cr ratio with lower GFR. There were positive correlations between urine protein/Cr, MCP-1/CCL-2/Cr, and netrin-1 with NGAL (r = 0.397, p = 0.031; r = 0.437, p = 0.041, r = 0.323, p = 0.042, respectively). Urine netrin-1/Cr was positively correlated with MCP-1/ CCL-2/Cr (r = 0.356, p = 0.045). There were positive associations between the presence of proteinuria and netrin-1/ Cr, MCP-1/CCL-2/Cr, and NGAL/Cr [Odds ratio (OR): 1.423, p = 0.037, OR: 1.553, p = 0.033, OR: 2.112, p = 0.027, respectively)]. ROC curve analysis showed that netrin-1/Cr, MCP-1/CCL-2/Cr, and NGAL/Cr had high predictive values for determining proteinuria p = 0.027, p = 0.041, p = 0.035, respectively). Urine hepcidin/ Cr was negatively correlated with tubular phosphorus reabsorption and was positively correlated with urine NGAL/Cr (r = -0.418, p = 0.019; r = 0.682, p = 0.000; respectively).

**Conclusions:**

MCP-1/CCL-2 may play a role in the development of proteinuria in MCDK. Netrin-1 may be a protective factor against proteinuria-induced renal injury. Urine hepcidin/Cr may reflect proximal tubule damage in MCDK. Urine NGAL/Cr may be a predictor of tubule damage by proteinuria.

## Resumo

**Introdução:**

A hiperfiltração glomerular pode causar proteinúria e doença renal crônica no rim displásico multicístico unilateral (RDM). Nosso objetivo foi investigar os níveis de lipocalina associada à gelatinase neutrofílica na urina (NGAL), netrina-1, hepcidina e quimiocina C-C com ligante-2 (MCP-1/CCL-2) em pacientes com RDM.

**Métodos:**

Trinta e dois pacientes e 25 controles foram incluídos. Os níveis urinários de hepcidina, netrin-1, NGAL e MCP-1/CCL-2 foram determinados por ELISA.

**Resultados:**

Os pacientes apresentaram níveis séricos mais elevados de creatinina (Cr), albumina na urina e relação netrina-1/Cr com menor TFG. Houve correlação positiva entre proteína na urina/Cr, MCP-1/CCL-2/Cr e netrina-1 com NGAL (r = 0,397, p = 0,031; r = 0,437, p = 0,041, r = 0,323, p = 0,042, respectivamente). A netrina-1/Cr na urina foi correlacionada positivamente com MCP- 1/CCL-2/Cr (r = 0,356, p = 0,045). Houve associações positivas entre a presença de proteinúria e netrina-1/Cr, MCP-1/ CCL-2/Cr e NGAL/Cr [Odds ratio (OR): 1,423, p = 0,037, OR: 1,553, p = 0,033, OR: 2,112, p = 0,027, respectivamente) ]. A análise da curva ROC mostrou que netrina-1/Cr, MCP-1/CCL-2/Cr e NGAL/ Cr apresentaram altos valores preditivos para determinar a proteinúria p = 0,027, p = 0,041, p = 0,035, respectivamente). A hepcidina/Cr na urina foi correlacionada negativamente com a reabsorção tubular de fósforo e positivamente com a NGAL/Cr na urina (r = -0,418, p = 0,019; r = 0,682, p = 0,000; respectivamente).

**Conclusões:**

MCP-1/CCL-2 pode ter participação no desenvolvimento de proteinúria no RDM. A Netrina-1 pode ser um fator protetor contra lesão renal induzida por proteinúria. Hepcidina/Cr na urina pode refletir danos em túbulos proximais no RDM. O valor de NGAL/Cr urinário pode ser um preditor de danos nos túbulos por proteinúria.

## INTRODUCTION

Children with a solitary functioning kidney (SFK) have an increased risk of developing kidney failure later in their lives. Unilateral multicystic dysplastic kidney (MCDK) is one of the most common causes of congenital SFK (cSFK). Glomerular hyperfiltration in the remnant nephrons due to decreased renal mass leads to glomerulosclerosis, hypertension, and proteinuria in the early period of life.^1^ Tubular injury has been proposed as the final common pathway for chronic kidney disease progression.^2^

The increased flow of the glomerular filtrate causes fluid shear stress (FSS) on the apical surface of the proximal tubule cells in the remnant nephron, while remnant nephron hypertrophy increases metabolic demand and causes epithelial tubular structural changes in the proximal tubular cells.^3^

Endothelial cells and kidney tubular epithelial cells secrete netrin-1, a laminin-related molecule. Netrin-1 has a molecular mass of 72 KDa, so it is not unlikely filtered by the glomerulus under normal conditions.^4^ Netrin-1 expression is induced 3 hours after ischemia-reperfusion in proximal tubular epithelial cells. Netrin-1 level attains peak level at 24 hours.^4^ It was shown that netrin-1 is expressed by proximal tubular epithelial cells, and is determined in the urine immediately after reperfusion. Thus, netrin-1 is thought to be an early diagnostic biomarker of acute kidney injury.^5^ Inflammation and apoptosis in the tubular epithelial cells are regulated by netrin-1 in acute kidney injury.^6^

Hepcidin is a low molecular weight peptide (2.78 kDa) produced by the liver. Hepatic production of hepcidin is increased by high iron levels and inflammation.^7^ Urine excretion of hepcidin is low in normal subjects.^8^ The increased urine hepcidin levels is thought to reflect the decreased proximal tubular reabsorption.^9^

The production of C-C motif chemokine ligand 2 (CCL-2, also known as monocyte chemoattractant protein-1 [MCP-1]), is a potent chemotactic factor for monocytes, and it also increases in response to proinflammatory cytokines during inflammation, and several studies have reported roles for MCP-1/CCL2 in renal diseases. MCP-1/CCL2 is associated with tubulointerstitial damage and interstitial fibrosis in IgA nephropathy.^10^ Significant associations have also been found between urine levels of MCP-1/CCL-2 and kidney MCP-1/CCL-2 expression in response to interstitial macrophage accumulation in diabetic nephropathy.^11^

Injured proximal tubular cells also secrete increased amounts of neutrophil gelatinase-associated lipocalin (NGAL), a member of the lipocalin superfamily, into the urine during ischemic or nephrotoxic kidney injury. NGAL is expressed in neutrophils but only at low levels in the normal kidney. Therefore, NGAL has been suggested as an early urinary biomarker for active kidney damage, in place of functional parameters such as serum creatinine or glomerular filtration rate (GFR).^12^

The exact mechanism of hyperfiltration-mediated injury remains unclear.^13^ It is considered that the changes associated with biomechanical forces within the glomerulus and cellular response to biomechanical forces in terms of hemodynamic parameters play a role in the development of injury from hyperfiltration.^14^ This paper describes a cross-sectional study involving the children with unilateral MCDK followed up in our department. The aim of the study was to investigate the urine NGAL, netrin-1, hepcidin, and MCP-1/CCL-2 levels in these patients at increased risk of renal injury caused by glomerular hyperfiltration. We also evaluated the associations between these potential biomarkers and proteinuria and GFR in our patients with MCDK.

## MATERIALS AND METHODS

### STUDY GROUP

This study is a single-center cross-sectional study. Participants were divided in two groups: patients with MCDK and healthy controls. The patients with MCDK were followed up in the Pediatric Nephrology Outpatient Clinic between September 2010 and March 2017. Unilateral MCDK was documented by renal ultrasound and dimercaptosuccinic acid scintigraphy.

In our Pediatric Nephrology Clinic, the diagnosis of urinary tract infection (UTI) was made based on the presence of at least 100.000 colony-forming units/mL of a uropathogen cultured from the urine specimen and pyuria (WBC count ≥5 as measured with a high-power field on a microscopic urinalysis) and UTI symptoms. Recurrent UTI (RUTI) was defined as two or more episodes of acute pyelonephritis or acute pyelonephritis plus one or more episode of cystitis or three or more episodes of cystitis.^15^ Hydronephrosis was defined using the Society for Fetal Urology’s grading system.^16^ Voiding cystourethrography (VCUG) was done only in children with RUTI or renal scarring on dimercaptosuccinic acid scintigraphy.

Patients who had signs and symptoms of infection, a history of UTI, or other kidney abnormalities (such as hydronephrosis or vesicoureteral reflux) were excluded from the study. Patients taking medication that might impair kidney function were also excluded from the study.

Age and gender-matched healthy children were included as a control group. Children with signs and symptoms of infection, a history of urinary tract infection, chronic inflammatory diseases, or kidney or urinary tract anomalies were excluded from the control group.

Peripheral venous blood samples were obtained in the morning after an overnight fasting. Serum creatinine (Cr), blood urea nitrogen (BUN), electrolytes, hemoglobin, serum iron and ferritin levels, iron-binding capacity, and transferrin saturation were determined from the blood specimens for each patient and control child. Any children with abnormal iron metabolism parameters were not included in this study because high iron levels could increase hepcidin levels. Morning urine samples were centrifuged, and the supernatants were frozen at −80 C° until further use. Tubular phosphate reabsorption (TPR), urinary excretion of albumin, creatinine, and protein were measured in morning samples. A spot urine albumin/creatinine ratio (ACR) of 30-300 mg/g was defined as microalbuminuria. Proteinuria was defined as a protein/creatinine ratio ≥ 0.2 mg/mg (more than 0.5 for children 6-24 months of age). Estimated glomerular filtration rate [eGFR (mL/min/1.73 m^2^) = k × body length (cm)/serum Cr level (mg/dL)] was determined by the old Schwartz formula.^17^

Urine NGAL, netrin-1, hepcidin, and MCP-1/CCL-2 concentrations were determined using enzyme-linked immunosorbent assay (ELISA) methods (Elabsience, Wuhan, China) with intra-assay and inter-assay coefficients of variation <10 % (Catalog number for NGAL kit: E-EL-H0096; netrin-1: E-EL-H2328; hepcidin: E-EL-H0077, and MCP-1/CCL-2: E-EL-H0020). The sensitivities of the ELISA kits were 0.1 ng/mL, 18.75 pg/mL, 0.1 ng/mL, and 9.38 pg/mL, respectively. As a heterogeneous assay, ELISA separates some components of the analytical reaction mixture by adsorbing certain components that are physically immobilized onto a solid phase. Absorbance readings and calculations were conducted using VICTOR X3 microplate reader (Perkin Elmer, Waltham, United States). The results are reported as netrin-1/creatinine (Cr) and MCP-1/CCL-2/Cr in pg/mL. The NGAL/Cr and hepcidin/Cr values are reported in ng/mL.

### ETHICS COMMITTEE APPROVAL

The procedures were approved by the local Ethical Committee Board (approval number 80558721/130). Informed consent was obtained from guardians or parents of each participant included in the study. The study protocol was consistent with the ethical guidelines of the 1975 Declaration of Helsinki as reflected in a prior approval by the institution’s human research committee.

### STATISTICAL ANALYSIS

Statistical analyses were performed using SPSS 11.5 (SPSS Inc, Chicago, IL). Values are reported as mean and SD for normally distributed continuous variables and median and interquartile range (IQR) for non-normally distributed continuous variables. The Shapiro-Wilk test was used to determine normality of data. Means were compared using the independent sample t-test for normally distributed data. Non-normally distributed data were compared using the Mann-Whitney U test. Correlations between variables were evaluated using Pearson’s or Spearman’s test, as appropriate. Qualitative variables were compared using the chi-square test. Linear regression was performed to explore the relationship between urinary biomarkers and eGFR as the dependent variable. A logistic regression analysis was performed to determine the influence of these urinary biomarkers on the presence of proteinuria and microalbuminuria in patients with MCDK. Receiver-operating characteristic (ROC) analysis was used to determine the cutoff values and the sensitivity/specificity of NGAL/Cr. A p value < 0.05 was considered statistically significant.

## RESULTS

**Figure 1:**
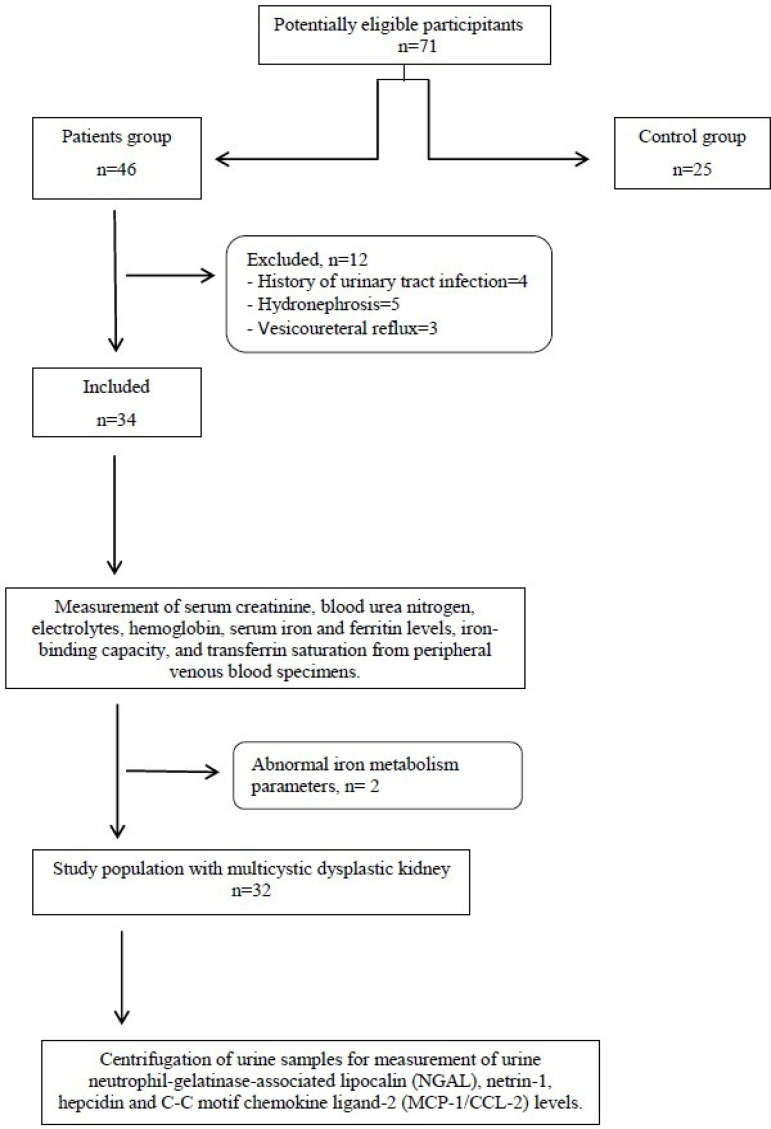
Flow-chart of patient selection.

Fourteen of 46 patients with MCDK in our pediatric nephrology clinic were excluded because they did not meet the criteria. Of the14 excluded patients, 4 had history of RUTI. Hydronephrosis was detected in 5 patients. Three of 4 patients who underwent VCUG had VUR. Two patients were excluded due to iron deficiency. In total, 32 patients with MCDK and 25 healthy children were included in this study. The description of participant recruitment flow in shown as a diagram in Figure 1.

The demographic and laboratory data of the patients and control group are shown in Table 1. Serum creatinine levels and ACR were higher in patients than in the control group [0.53 ± 0.23 versus 0.4 ± 0.13 mg/dL, p = 0.031; 11.4 (4.6-23.72) versus 6.35 (2.92-10.9) mg/g, p = 0.037, respectively]. The eGFR was lower in the patients than in the controls (140.6 ± 28.33 versus 175.8 ± 29.69 mg/dL, respectively, p = 0.000). The urine netrin-1/Cr ratio was higher in patients than in the controls (p = 0.041).

**Table 1:** Laboratory and demographic data of the patients and controls.

	Patients (n = 32)	Control group (n = 25)	p
Age (years)	7.5 (3.25-13)	8 (5-9.5)	0.881
Female gender (n, %)	19 (59.4%)	9 (36%)	0.082
Phosphorus (mg/dL)	4.9 ± 0.89	5 ± 0.76	0.561
Creatinine (mg/dL)	0.53 ± 0.23	0.4 ± 0.13	0.031
TPR (%)	94.6 ± 3.98	94.3 ± 2.66	0.213
ACR (mg/g)	11.4 (4.6-23.72)	6.35 (2.92-10.9)	0.037
Protein-to creatinine ratio	0.18 (0.12-0.33)	0.15 (0.11-0.18)	0.511
GFR (mL/min/1.73 m^2^)	140.6 ± 28.33	175.8 ± 29.69	0.000
Urine netrin-1(pg/mg creatinine)	0.26 (0.13-0.55)	0.14 (0.11-0.21)	0.041
Urine hepcidin (ng/mg creatinine)	0.35 (0.26-0.67)	0.45 (0.25-0.56)	0.502
Urine MCP-1/CCL-2 (pg/mg creatinine)	0.13 (0.04-0.33)	0.08 (0.03- 0.24)	0.543
Urine NGAL (ng/mg creatinine)	0.2 (0.13-0.36)	0.22 (0.13-0.35)	0.672

Values are reported as mean ± SD or median (interquartile range). TPR: tubular phosphate reabsorption; ACR: albumin-to creatinine ratio; GFR: glomerular filtration rate, MCP / CCL: monocyte chemoattractant protein / C-C motif chemokine ligand; NGAL: neutrophil gelatinase-associated lipocalin. A p value< 0.05 was considered significant.

**Figure 2:**
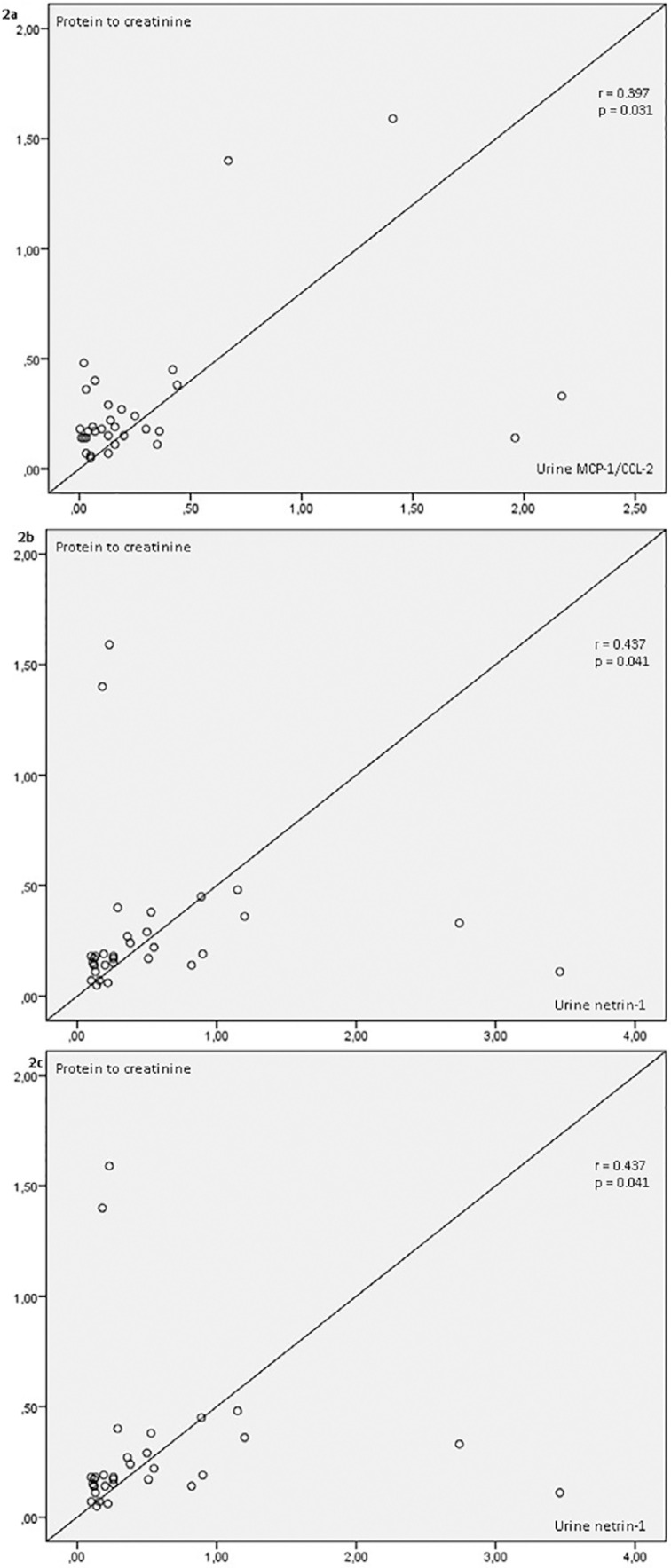
(a) Correlation between protein to creatinine and MCP-1/CCL2, (b) Correlation between protein to creatinine and urine netrin-1, (c) Correlation between urine netrin-1 and MCP-1/CCL2.

**Figure 3:**
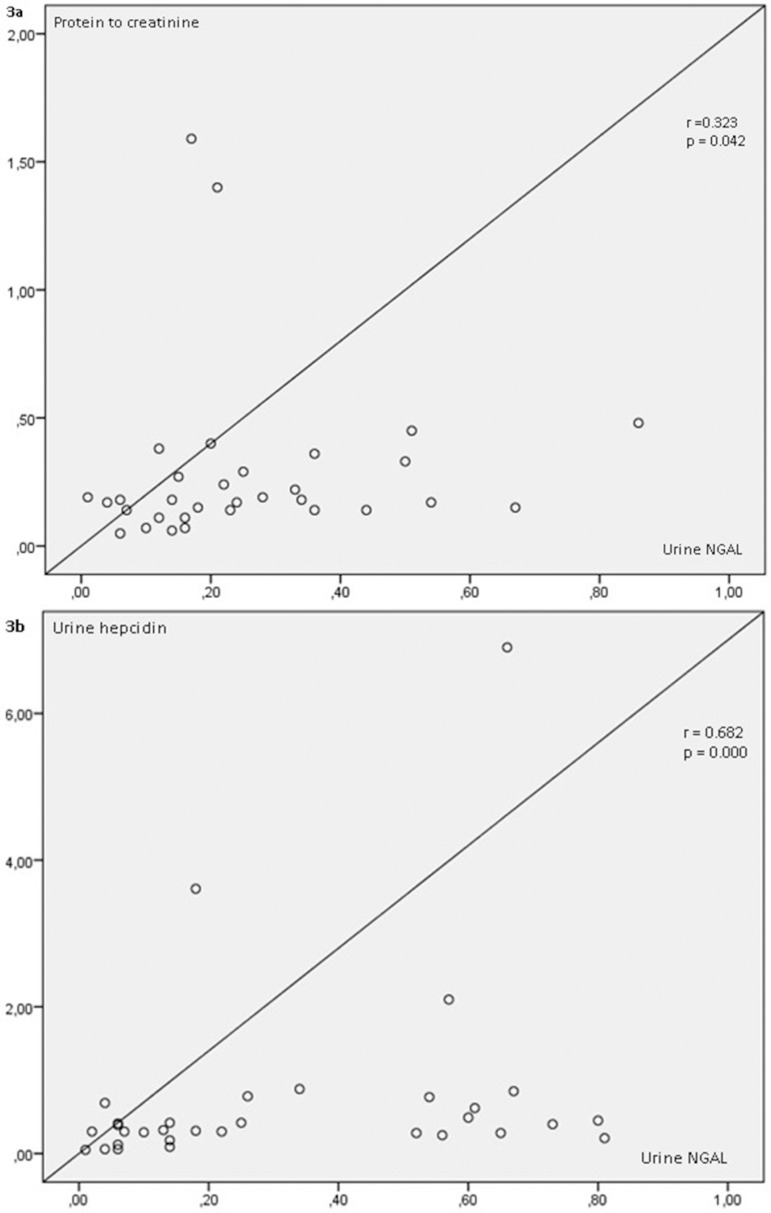
(a) Correlation between protein to creatinine ratio and urine NGAL. (b) Correlation between urine hepcidin and NGAL.

The correlation analysis showed a negative correlation between the spot urine protein/Cr ratio and the %TPR (r = -0.43, p = 0.003). Significant positive correlations were detected between spot urine protein/Cr and urine MCP-1/CCL-2 with netrin-1/Cr (r = 0.397, p = 0.031; r = 0.437, p = 0.041, respectively, Figure 2a and 2b). A significant positive correlation was also found between urine netrin-1/Cr and urine MCP-1/CCL-2/Cr (r = 0.356, p = 0.045, Figure 2c). Urine NGAL/Cr was positively correlated with spot urine protein/Cr and urine hepcidin/Cr (r = 0.323, p = 0.042; r = 0.682, p = 0.000, respectively, Figure 3a and 3b). Urine hepcidin/Cr was negatively correlated with %TPR (r = -0.418, p = 0.019). GFR was not correlated with ACR or the urinary protein-to-creatinine ratio (r = 0.07, p = 0.704; r = -0.016, p = 0.931, respectively).

**Figure 4:**
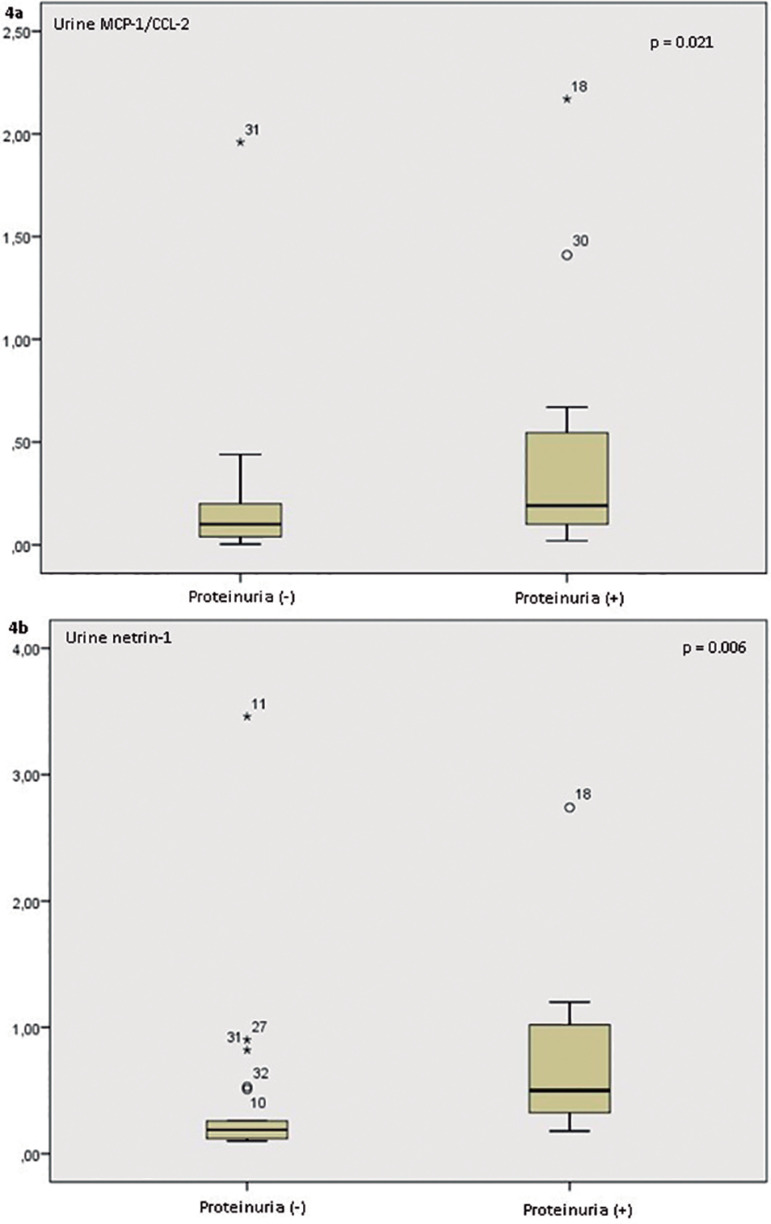
(a) The patients with proteinuria had higher urine MCP-1/ CCL2 levels and (b) higher urine netrin-1 levels.

Eleven patients (34.4%) had proteinuria. Comparison of the laboratory data of patients with proteinuria and non-proteinuric patients revealed higher urine NGAL/Cr levels in the proteinuric than in the non-proteinuric patients [0.22 (0.19-0.42) versus 0.17 (0.09-0.34) ng/mg creatinine, respectively, p = 0.034]. Urine MCP-1/CCL-2 and netrin-1 ratios were higher in proteinuric patients than in non-proteinuric patients [0.29 (0.26-0.32) versus 0.12 (0.05-0.21) pg/mg creatinine, p = 0.021; 0.5 (0.29-1.15) versus 0.19 (0.12-0.38) pg/mg creatinine, p = 0.006, respectively, Figure 4a and 4b).

ROC curve analysis showed that the cut-off value of urine NGAL/Cr for the prediction of proteinuria was 0.27 ng/mg creatinine, with a sensitivity of 91.1% and a specificity of 95.1%. The area under the curve (AUC ± SE) was 0.729 ± 0.088 [95% confidence interval (CI): 0.558-0.901, p = 0.035]. The ROC curve analysis revealed that urine MCP-1/CCL-2/Cr had a high predictive value for determining proteinuria, with a sensitivity of 74.1% and a specificity of 65.8% (AUC ± SE: 0.654 ± 0.072, cut-off value: 0.24 pg/mg creatinine, 95% CI: 0.608-0.831, p = 0.041). Urine netrin-1/Cr was a predictor for proteinuria, with a sensitivity of 81.8% and a specificity of 71.4% (AUC ± SE: 0.797 ± 0.079, 95% CI: 0.643-0.950, cut-off value: 0.185 pg/mg creatinine, p = 0.027).

The factors associated with the presence of proteinuria in patients were investigated by logistic regression analysis. Significant positive associations were found between the presence of proteinuria and urine netrin-1/Cr, MCP-1/CCL-2/Cr, and NGAL/Cr [odds ratio (OR): 1.423, p = 0.037, OR: 1.553, p = 0.033, and OR: 2.112, p = 0.027, respectively). The detailed results are shown in Table 2.

**Table 2:** Logistic regression analysis evaluating factors associated with presence of proteinuria in patients.

	Odds ratio	95% CI	p
Serum creatinine	0.539	0.304 - 1.690	0.487
eGFR	0.748	0.618 - 0.999	0.867
Netrin-1	1.423	1.243 - 4.823	0.037
Hepcidin	0.568	0.849 - 3.663	0.128
MCP-1/CCL-2	1.553	1.386 - 6.432	0.033
NGAL	2.112	2.105 - 8.225	0.027

CI: confidential interval; eGFR: estimated glomerular filtration rate; MCP / CCL: monocyte chemoattractant protein / C-C motif chemokine ligand; NGAL: neutrophil gelatinase-associated lipocalin. A p value< 0.05 was considered significant.

## DISCUSSION

We investigated urine hepcidin, netrin-1, NGAL, and MCP-1/CCL-2 levels in children with MCDK. Our results revealed that the patients with MCDK had a higher urine netrin-1/Cr ratio when compared with healthy controls. The urine MCP-1/CCL-2/Cr, netrin-1/Cr, and NGAL /Cr ratios showed significantly associations with the presence of proteinuria.

Compensatory hypertrophy in the remnant kidney contributes to albuminuria and a decreased GFR. Microalbuminuria is considered a manifestation of glomerular vascular endothelium damage. The larger amount of albumin filtered because of glomerular injury may exceed the albumin reabsorption capacity of the tubules. Another possibility is that the changes in tubular albumin reabsorption may play a role in the development of microalbuminuria.^18^

The current literature shows conflicting results regarding the association between glomerular hyperfiltration and microalbuminuria in children. For example, Schreuder et al. showed that there was microalbuminuria in 23% of 66 patients with cSFK.^19^ By contrast, Cachatet al. investigated the association between microalbuminuria and filtration fraction in SFK and found very poor association in patients with normal GFR.^20^ In the present study, we detected microalbuminuria in half of our patients, but were unable to find a significant association between microalbuminuria and GFR. This result might support the possibility that microalbuminuria is caused by proximal tubular dysfunction or other unidentified factors in children with MCDK.

Renal function measurement is often focused on the GFR. The determination of the real value is quite troublesome, expensive, and difficult in daily practice. Therefore, the use of estimated GFR value is recommended as an alternative method.^21^^,^ 22

The measurement of eGFR value depends on serum creatinine level, which reflects muscle mass. Muscle mass has wide variation among individuals.^23^ Furthermore, several studies described hyperfiltration as the result of increased capillary pressure in glomeruli.^24^^,^ 25 In our study, eGFR was significantly higher the in control group. We used eGFR based on serum creatinine level. In addition, we did not evaluate muscle mass in our study group. This result may be due to individual differences among children.

Proteinuria is viewed as an important predictor of the activity and progression of kidney diseases. Proteinuria itself can also lead to the progression of kidney damage and reduction in GFR in patients with reduced nephron mass. Thus, therapeutic interventions for reducing proteinuria are suggested to slow the progression of chronic kidney disease and the reduction in GFR.^26^ Several experimental studies have shown a positive association between albuminuria and tubular MCP-1/CCL-2 expression.^27^^,^ 28 MCP-1/CCL-2, which is produced by mesangial and tubular epithelial cells, is expressed by activated monocyte/macrophages, T cells, and natural killer cells. MCP-1/CCL-2 plays an important role in leukocyte infiltration into the kidney and in the development of tubulo-interstitial fibrosis.^29^ The inhibition of MCP-1/CCL2 overproduction caused by albumin exposure was shown to restore podocyte dysfunction in rats.^30^ The current literature shows significant correlations between urine MCP-1/CCL-2 and protein levels in patients with lupus nephritis or primary glomerulonephritis.^31^^,^ 32 Several studies have also evaluated urine MCP-1/CCL2 levels in kidney diseases in children. For example, Wanget al. revealed that urine MCP-1/CCL-2 levels were associated with proteinuria, but not with serum Cr and BUN levels in children with Henoch-Schonlein purpura nephritis.^33^ Wasilewska et al. suggested that permanent proteinuria and progressive kidney fibrosis could lead to increased urine MCP-1/CCL-2 levels in children with glomerular proteinuria.^34^ Bartoli et al. showed that urine MCP-1/CCL2 levels were significantly higher in children with MCDK when compared with the control group. They suggested that chronic renal inflammation by local monocytes is a main factor in the development of progressive renal damage.^35^ In our study, we found a significant association between urine MCP-1/CCL-2 and proteinuria, but not with GFR or serum creatinine. The higher urine MCP-1/CCL-2/Cr ratio might be caused by proteinuria in children with MCDK. Therefore, the urine MCP-1/CCL-2/Cr ratio might be a predictor for GFR-independent proteinuria.

Netrin-1, which has a molecular mass of 72 KDa, is not filtered by the glomerulus under normal conditions. Thus, an effect of netrin-1 on glomeruli is difficult to detect. Healthy tubular epithelial cells do not express or express only low levels of netrin-1. During ischemic injury, netrin-1 produced by proximal tubular epithelial cells is secreted into the tubule lumen and is excreted in urine.^36^ Recent studies further indicate that high urine netrin-1 levels may be a biomarker for early detection of acute kidney injury.^37^ Urine netrin-1 levels are known to increase in chronic kidney disease, as well as following acute kidney injury.^4^ Li et al. revealed that urine netrin-1 levels were higher in children with an obstructed kidney than in a non-obstructive hydronephrosis group and in healthy children.^38^ We demonstrated a higher urine netrin-1/Cr in patients with MCDK than in our healthy control group. Also, urine netrin-1/Cr was positively associated with the presence of proteinuria in our patients. These findings suggest that urine netrin-1 may be increasing to protect epithelial cells from harmful effects of proteinuria.

Proteinuria can play an important role in the development of tubulo-interstitial injury and in the decrease in renal function in the long term. The increased reabsorption of filtered proteins can lead to proximal tubular damage.^39^ Conversely, proximal tubular damage may lead to clinical proteinuria due to impaired tubular endocytosis of albumin.^40^ NGAL is a biomarker of tubular damage caused by the changes in fluid shear stress on proximal tubular cells.^41^ In our study, proteinuric patients had a higher urine NGAL/Cr ratio when compared with non-proteinuric patients. Proximal tubule epithelial cell damage might therefore lead to proteinuria in patients with MCDK, and vice versa. The interaction between the reabsorption of NGAL at the proximal tubule with the increased filtered albumin could increase urinary NGAL excretion in proteinuric patients.^42^ The increased levels of urine NGAL therefore probably reflect the tubule damage by proteinuria.

Hepcidin, which is a regulator protein of iron homeostasis, is freely filtered through the glomerulus. Hepcidin is both reabsorbed by proximal tubule epithelial cells and synthesized in the distal tubule region.^43^ Recent studies have indicated that urine hepcidin level might be a potential biomarker for acute kidney injury.^44^ For example, Fufaa et al. demonstrated that urine hepcidin level could serve as a potential biomarker of inflammatory cell invasion in early diabetic nephropathy lesions.^45^ In our study, we did not find elevated urine hepcidin levels in our patients, but we did find a positive correlation between urine hepcidin/Cr and NGAL/Cr levels. In addition, the urine hepcidin/Cr ratio was negatively correlated with the TPR values. The urine hepcidin/Cr ratio might therefore reflect decreased proximal tubular reabsorption due to epithelial cell damage in patients with MCDK.

Our study has some limitations. It was a cross-sectional study with a small sample size. We did not measure the serum levels of the indicated potential biomarkers, so we could not determine the relationship between urine and serum levels. We did not collect 24-hour urine samples for measurements. The true GFR was not measured in our study population. In addition, we did not assess middle and long-term effects of the potential biomarkers. Nevertheless, this is the first study to investigate the clinical significance of NGAL, netrin-1, and hepcidin in the urine of children with MCDK.

## CONCLUSION

The results of our study suggest that MCP-1/CCL-2 may play a role in the development of proteinuria in MCDK, while netrin- 1 may be a biomarker indicating the presence of proteinuria. Urine hepcidin may reflect proximal tubule damage in children with MCDK. Urine NGAL/Cr ratio may be a predictor of tubule damage by proteinuria. Prospective studies with larger sample sizes are needed to confirm whether these biomarkers are associated with glomerular or proximal tubular damage in patients with MCDK.
